# Clinical Characteristics and Surgical Outcomes in Patients With Intermittent Exotropia: A Large Sample Study in South China

**DOI:** 10.1097/MD.0000000000002590

**Published:** 2016-02-08

**Authors:** Min Yang, Jingchang Chen, Tao Shen, Ying Kang, Daming Deng, Xiaoming Lin, Heping Wu, Qiwen Chen, Xuelian Ye, Jianqun Li, Jianhua Yan

**Affiliations:** From the State Key Laboratory of Ophthalmology, Zhongshan Ophthalmic Center, Sun Yat-sen University, Guangzhou, The People's Republic of China.

## Abstract

The clinical characteristics and surgical outcomes in a large sample of patients with intermittent exotropia (IXT) as well as an analysis of risk factors associated with surgical failures are presented in this article. Data from IXT patients who received surgical management at the Eye Hospital, in the Zhongshan Ophthalmic Center, of Sun Yat-Sen University, China from January 2009 to December 2013 were reviewed retrospectively. Included within this analysis were data from pre- and postoperative ocular motility, primary alignment, and binocular vision.

A total of 1228 patients with IXT were reviewed. Males (50.4%) and females (49.6%) were nearly equally represented in this sample. Thirty-two patients (2.6%) had a family history of strabismus. The mean age at onset was 6.77 ± 6.43 years (range 7 months –48.5 years), mean duration at presentation was 7.35 ± 6.68 years (range 6 months–47 years), and mean age at surgery was 13.7 ± 8.8 years (range 3–49 years). The mean refractive error was −0.84 ± 2.69 diopter in the right eye and −0.72 ± 2.58 diopter in the left eye. Amblyopia (4.2%), oblique muscle dysfunction (7.0%), and dissociated vertical deviation (4.7%) were also present in these patients. The most common subtype of IXT was the basic type (88.1%). Orthophoria was observed in 80.5% of patients and the ratios of surgical undercorrection and overcorrection were 14.7% and 4.8%, respectively, as determined with a mean follow-up time of 7.8 ± 3.7 months. When combining ocular alignment with binocular vision as the success criteria, the success rate decreased to 35.6%. Multivariate risk factor analysis showed that only the loss of stereoacuity (*P* = 0.002) was associated with a poor outcome. There were no differences in the long-term results between bilateral lateral rectus recession and unilateral lateral rectus recession with medial rectus resection.

Most IXT patients displayed normal vision, with few having positive family histories, amblyopia, oblique muscle dysfunction, and dissociated vertical deviation. The most common subtype of IXT was the basic type. Long-term surgical results were less favorable when sensory status was included in the criteria for success. Patients with stereoacuity loss were at an increased risk for poor outcomes.

## INTRODUCTION

Intermittent exotropia (IXT) is the most common type of exotropia with an occurrence of approximately 0.5% to 1% in the general population. This condition accounts for approximately 25% of strabismus in children in the Western world^1,2^ and 44.9% of children in China.^3^ The primary symptom of IXT is an intermittent outward deviation involving either 1 or both eyes, especially while staring at distant objects in sunlight or when fatigued. The natural history of IXT remains unknown with reports indicating that this condition can either maintain its status for an extended period of time or show progressive deterioration.^4^ Precise clinical indications for strabismus surgery have yet to be determined, although patients showing extensive deviations, poor ocular control, and loss of binocular vision should be considered for surgical correction.^4–6^ Success rates of such surgeries are highly variable ranging from 38% to 91.6%, depending on the follow-up periods and the criteria used for determining success^7–11^; nor have definitive risk factors for poor surgical outcomes been clearly identified.^6,7^ While there are a number of studies on IXT in the literature, the small sample sizes in these reports make it difficult to reliably interpret the results. Therefore, a clear need exists for further studies with a large sample size to provide more reliable evidence for its management. Currently, only a few studies include sensory status in the evaluation of surgical outcomes^6^ and large sample studies assessing surgical outcomes of IXT are rare.^11–14^ Some examples of these studies include Yang et al,^11^ where only the motor status was used as a successful criteria. Other studies have been directed at identifying factors that show a predisposition for esotropia^12,13^ or the risk factors for recurrence after surgery.^14^ The present study was designed to analyze the clinical characteristics and surgical outcomes of patients with IXT based on a large sample size. Included within our analyses were both the motor and sensory status for determinations of final outcomes along with an evaluation of the risk factors that may affect the success of surgery.

## PATIENTS AND METHODS

The medical records of 1228 patients with IXT subjected to surgical correction while under general anesthesia at the Eye Hospital of the Zhongshan Ophthalmic Center, of Sun Yat-Sen University, China, from January 2009 to December 2013 were reviewed retrospectively. Written informed consent to participate in this study was obtained from all patients. Institutional approval for this study was obtained from the Research Ethics Board of the Zhongshan Ophthalmic Center, of Sun Yat-sen University, China, and all procedures were performed in accordance with the 1964 Declaration of Helsinki. The following clinical characteristics were recorded from the patients’ charts: family history, age at onset, age at surgery, best corrected visual acuity, cycloplegic refraction, preoperative motor alignment at distance and near, stereoacuity at distance and near, presence of an A or V pattern, presence of dissociated vertical deviation (DVD), and surgical methods employed. Patients with constant exotropia on presentation, neurological deficits, coexistent restrictive or paretic strabismus, or previous strabismus surgery were excluded. The decision to operate was determined by agreement of preferences expressed by the surgeon and patient, with indications for surgery including suppression and abnormal retinal correspondence by major amblyoscope, progressive loss of stereopsis, weaker control of exodeviation, and progressive increase in the angle of exotropia. All subjects in our study were first surgery candidates. We usually employed bilateral lateral rectus recession (BLR) for divergence excess or pseudodivergence excess, and unilateral lateral rectus recession with medial rectus resection (RR) for patients with a dominant eye deviation. For basic IXT, either BLR or RR was used. Medial rectus resections were used for convergence insufficiency, while a unilateral lateral rectus recession was used in patients with a maximum deviation angle of 25 prism diopter (PD). Surgeries involving 3 horizontal muscles were the preferred technique for exotropia greater than 60 PD. In general, strabismus surgery was performed under conditions based on the maximal angle of exotropia as measured preoperatively. Briefly, for the basic type of exotropia, we chose the largest angle measured at distance or at near for our surgical plan. For convergence insufficiency and pseudo-divergence excess types, we chose the largest angle at near and at distance, respectively. All of the surgeries were performed by 4 strabismus surgeons and all study examinations were conducted by 1 of 3 coauthors. Briefly, ocular alignment was assessed with the use of cover/uncover and alternate prism cover testing at distance (6 m) in primary position and in the cardinal gaze positions. Motor alignment at near was assessed at 33 cm with the use of spectacle correction. Stereoacuity at far was measured by random-dot stereograms and at near by Titmus stereograms. Hyperopic anisometropia was classified as the refractive difference between 2 eyes greater than 1 D, myopic anisometropia greater than 2 D, and astigmatic anisometropia greater than 1.5 D.^15^

Deviations of 8 PD or less of exotropia or 6 PD or less of esotropia were considered to be orthophoria. Overcorrection was defined as esotropia/phoria more than 6 PD and undercorrection as exotropia/phoria more than 8 PD.^16^ Stereoacuity less than 60 seconds both at far and near was considered normal. Surgical success rates were determined as based upon results obtained with the use of both motor and sensory criteria. Patients with orthophoria and normal stereopsis at far and near as determined at a minimum of 6 months follow-up were considered as surgical successes. Patients failing to meet these criteria for surgical success were considered as surgical failures (poor outcome). After classifying patients within 1 of these 2 groups (surgical success vs surgical failure), an attempt was made to identify preoperative risk factors for poor outcomes. Potential risk factors included sex, age at onset, age at surgery, preoperative symptoms and signs of amblyopia, anisometropia, oblique muscle dysfunction and dissociated vertical deviation, preoperative fusion and stereopsis, preoperative deviation and surgical methods.

The data were analyzed using SPSS software for Windows (version 17.0, Chicago, IL). Comparisons of risk factors for quantitative data of the surgical success versus surgical failure groups (including age at surgery, age at onset, duration of deviation, refractive errors, prism diopter at distance, and anisometropia) were analyzed by the Student *t* test or Mann–Whitney *U* test. Chi-square or Fisher exact tests were used for qualitative data (including sex, residual stereopsis, residual fusion, DVD, oblique muscle overaction, amblyopia, and surgery method). Multivariate logistic regression was used to analyze associations between risk factors and surgical outcomes. A *P* ≤ 0.05 was considered to be statistically significant.

## RESULTS

### Clinical Characteristics

A summary of the clinical characteristics for the 1228 patients with IXT included in this study is presented in Tables 1 and 2. Of these 1228 patients, the male-to-female ratio was nearly 1:1. A family history of strabismus was present in only 2.6% of the patients. The mean age at onset was 6.77 ± 6.43 years (range 7 months–48.5 years) and the mean age at surgery was 13.7 ± 8.8 years (range 3–49 years). The mean duration from onset of deviation to surgery was 7.35 ± 6.68 years (range 6 months–47 years). The mean refractive errors at their initial examination were −0.84 ± 2.69 D in the right eye and −0.72 ± 2.58 D in the left eye. Anisometropia was observed in 214 patients (17.4%) and amblyopia in 51 patients (4.2%). The amblyopia was due to anisometropia and a high refractive error. The incidence of other notable conditions included oblique muscle overaction (7.0%), dissociated vertical deviation (DVD, 4.7%), dissociated horizontal deviation (DHD, 0.2%), nystagmus (0.5%) and ptosis (0.2%). The most frequent pattern of exotropia was basic (88.1%), followed by pseudo-divergence excess (7.4%) and convergence insufficiency (4.5%). None of the patients in our study showed a true divergence excess type of exotropia. The mean preoperative angle of deviation of our patients was 41 ± 14 PD at distance and 40 ± 16 PD at near. Neither lateral incomitancy nor normal preoperative stereoacuity was observed in any of our patients. A small number of patients showed preoperative residual fusion (13.8%), stereoacuity (10.9%), or a combination of residual fusion and stereoacuity (5.9%) as determined by major amblyoscope. RR was the most commonly performed surgical procedure in our patients (55.7%), followed by BLR (23.6%), 3 horizontal muscle surgeries (9.8%), 1 horizontal rectus (8.6%), and binocular medial rectus resection (2.4%).

**TABLE 1:**
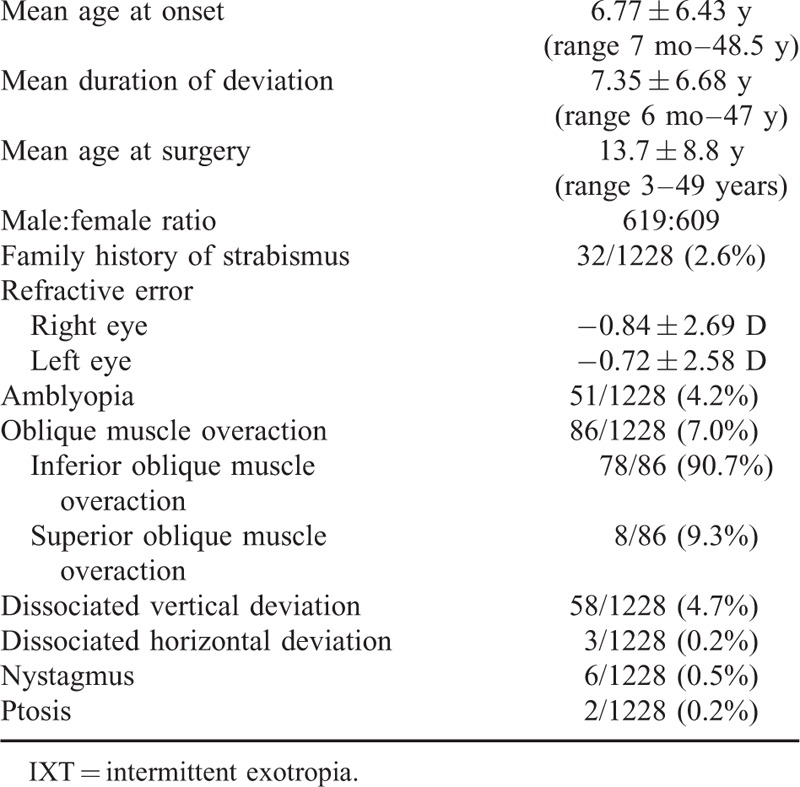
General clinical characteristics of IXT patients

**TABLE 2:**
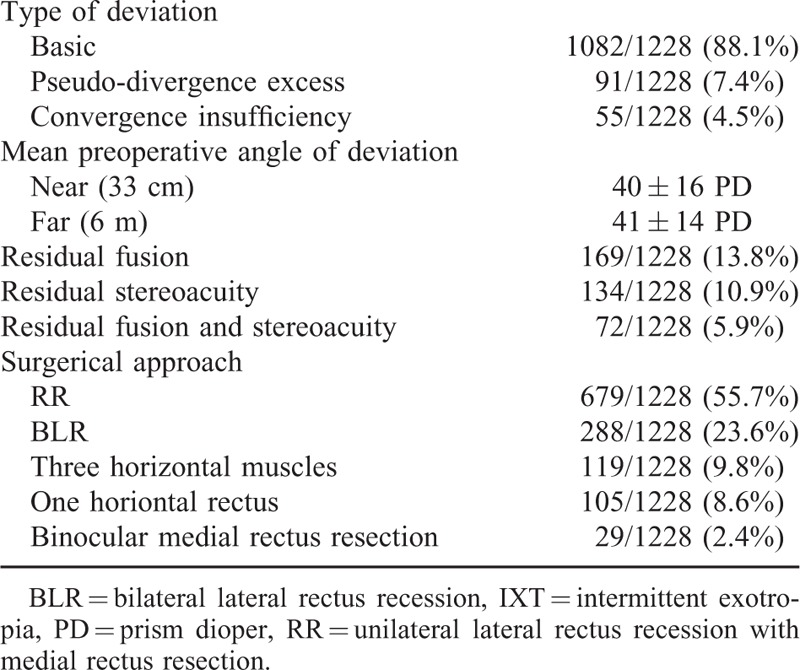
Characteristics of motor and sensory criteria and surgical methods in IXT patients

### Surgical Success Based on Motor and Sensory Criteria

Of the 1228 patients studied, 884 (72%) were followed up for an average period of 7.8 ± 3.7 months (range 6–23 months) after strabismus surgery. For patients classified as surgical successes based solely on the motor criteria, 712 (80.5%) were considered as demonstrating a postoperative surgical success, while 172 (19.5%) a surgical failure. A surgical undercorrection was observed in 14.7% (130/884) and an overcorrection in 4.8% (42/884) of the patients. When classified into surgical success based on both their motor and sensory status, the success rate decreased markedly to 35.6% (315/884).

### Risk Factor Analysis for Poor Outcomes

Univariate analysis revealed that preoperative residual fusion (*P* < 0.001), stereoacuity (*P* < 0.001), DVD (*P* = 0.004), amblyopia *(P = *0.02), and oblique muscle overaction (*P* = 0.006) were significantly associated with final surgical outcomes. Patients with a loss of fusion and stereoacuity, DVD, amblyopia, and oblique muscle overaction had poor outcomes. However, multivariate analysis revealed that residual stereoacuity (*P* = 0.002) was the only significant factor associated with final outcomes, with patients lacking residual stereoacuity demonstrating poor outcomes. Several variables such as sex, age at onset, age at surgery, duration of exodeviation, amblyopia, DVD, oblique muscle overaction, refractive errors, anisometropia, residual fusion, preoperative angle of exotropia, and surgical method employed showed no significant associations with final outcomes (Table 3).

**TABLE 3:**
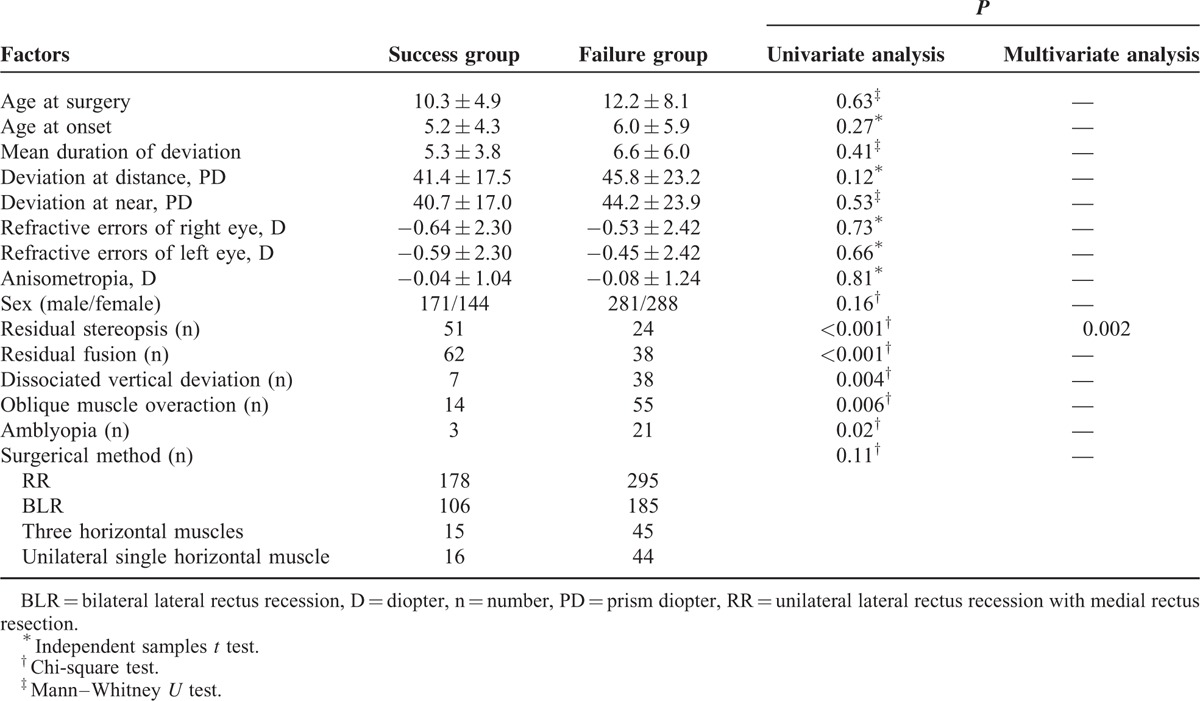
Preoperative risk factors for poor outcomes

## DISCUSSION

### Clinical Characteristics of Surgical Patients With IXT

IXT is the most prevalent type of exotropia^1^ and has been reported to be more prevalent in females.^17^ However, the number of males and females were nearly equal in our study, similar to that of Park.^18^ Only 2.6% of the patients had a positive family history of IXT in present study, which is consistent with a previous report.^18^ Most patients were referred with an intermittent outward deviation of 1 or both eyes, which often appeared when gazing at distant objects in sunlight or when fatigued and the frequency of this deviation showed a progressive increase within months following their initial symptoms. The most common subtype of IXT was the basic type, which is in accord with another study.^7^ The average refractive errors in our patients included a mild myopia. The best correct visual acuity in most patients was normal, but was affected in some subjects.^4^ In our study, amblyopia was observed in 4.2% of the patients, which was similar to that reported by Mohney,^19^ and anisometropia was the most common cause of amblyopia.

IXT may be associated with other motor anomalies, such as oblique muscle overaction, DVD, and DHD. Among these, oblique muscle overaction is the most frequent and may lead to an A-V pattern and small vertical deviation. Wilson et al^20^ reported that 32% of their patients showed oblique muscle dysfunction, which was considerably higher than the 7.0% in our study. When oblique muscle overaction and vertical deviation are mild and slight, surgical correction may not be required as these may improve after horizontal muscle surgery only.^21^ Struck et al^21^ reported a satisfactory outcome was obtained with horizontal muscle surgery alone in all 17 of their IXT cases with a mean 8 PD hypertropic deviation. In our cohort, oblique muscle surgery was performed if patients presented an obvious A or V pattern, their oblique muscle overaction was more than 2+, or their vertical deviation was more than 10 PD. In most studies, patients with an obvious vertical deviation, especially DVD, were excluded.^8,9,22^ However, DVD was included in our cohort and revealed another coincident common finding of our patients with a proportion of 4.7%, a rate that was similar to the 4.0% in the study of Jeoung.^23^ Similar to that for oblique muscle overaction, surgery for DVD was performed only in patients with vertical deviation more than 10 PD and a frequent occurrence of a remarkable updrift as observed by the patients or their parents. Twenty patients with a mean vertical deviation of 12.4 ± 0.3 PD were subjected to surgery for DVD in our study.

### Surgical Success and Risk Factor Analysis for Poor Outcomes

Many questions and unresolved issues exist regarding the natural history and surgical management of IXT. Although surgery is considered to be an effective method for correction of exotropia and restoration of binocular vision, there remains a disappointingly high rate of recurrent exodeviations or consecutive esodeviations. Classical surgical procedures for IXT include BLR and RR. BLR is the preferred approach for the divergence excess type, but controversy exists for this basic type. Yang et al^11^ reported that BLR showed a better success rate than RR (60.75% vs 43.40%) after an average of 3 years follow-up in study consisting a 50 cases; however, Wang et al^8^ stated that RR was more effective than BLR (85.1% vs 65.8% success rate) in a study including 85 patients with the basic type of IXT as determined at a mean follow-up of 14.8 ± 9.5 months. In general, there appears to be no significant difference between these 2 surgical procedures for IXT,^9^ which is consistent with our study relating success rates of 36.7% for RR and 37.9% for BLR. However, it should be noted that we did not classify IXT by its subtypes when patients were evaluated. Success rates of surgery for IXT can be quite variable ranging from 38% to 91.6%, because of differences in surgical approaches, standards used in evaluating success, and variations in follow-up times.^7–11^ At present, most researchers regard motor status as providing a criterion for success,^8–10^ while success rates would be much lower if sensory status was taken into account. In a study with a minimum postoperative follow-up of 10 years, Pineles et al^7^ found that 64% of their patients had an excellent outcome when only the motor criteria were used, but this classification of an excellent outcome decreased to 38% when a combined motor/sensory criteria for surgical success was used. Ekdawi et al^24^ reported a similar success rate of 45% after an 8-year follow-up when both motor and sensory criteria were used. In our study, which involved a relatively short average follow-up of 7.8 months, a satisfactory motor outcome was obtained in 80.5% of patients. However, in patients where both the motor and sensory criteria were used, success rates were only 35.6%, values that were lower than that of Ekdawi's report,^24^ but quite similar to the results of Pineles.^7^ It is also important to note that, because of the tendency of exotropic drift over time, success rates for surgical interventions in IXT decrease as postoperative follow-up time increase.^7,24,25^ Therefore, although widely studied, no universal evaluating criteria or standard treatment protocols for IXT have yet to be established. In the opinion of Chiu,^26^ a comprehensive assessment of surgical management of IXT in future studies should incorporate not only sensory and motor criteria, but also control outcomes and descriptions/opinions of patients/parents as a means to achieve an overall assessment of their health-related quality of life.

A major concern associated with poor surgical outcomes involves recurrent exodeviations that are mainly caused by the tendency for postoperative exodrift^25^ and can become more serious over time.^24^ The dosage of surgery required for the maximal amount of exotropia as detected presurgically and to achieve a controlled 20 PD overcorrection in the initial postoperative phase are the accepted clinical protocols for the treatment of exotropic drift.^27^ Results from a previous study have suggested that the initial postoperative deviation was considered as the only prognostic factor for success in the treatment of IXT^28^; however, others have reported little effect of initial postoperative deviation on surgical outcomes.^22,29,30^ Moreover, constant esotropia resulting from overcorrecton may damage binocular vision and lead to amblyopia. Considering the instability of presurgical exodeviations and tendency for postsurgical exodrift, we performed a more extensive surgical procedure in our patients to obtain a mild overcorrection in the initial postoperative phase. However, patients younger than 4 years should show either no or less overcorrection in the initial postoperative phase as compared with that of older children. Surgical undercorrection was observed in 14.7% of the patients in our study, which was lower than that of other studies^9,10^ and the proportion of surgical overcorrection was also lower than that of a previous report.^8^ Pineles et al^7^ reported that patients with anisometropia, lateral incomitance, and immediate postoperative undercorrection are at an increased risk for poor outcomes and require additional surgeries. The results of our univariate analyses indicated that factors such as loss of fusion, stereoacuity, DVD, amblyopia, and oblique muscle overaction were associated with poor surgical outcomes. Other factors such as sex, age at onset, age at surgery, duration of deviation, refractive errors, anisometropia, preoperative angle of exotropia, and surgical methods were not associated with surgical results. However, multivariate analysis revealed that residual stereoacuity was the only significant factor associated with final outcomes. Patients with preoperative residual stereoacuity were more likely to have favorable surgical outcomes. In our opinion, residual stereoacuity may aid in controlling both mild exodeviation (surgical undercorrection) and esodeviation (surgical overcorrection), thereby enhancing the potential for restoring binocular vision after strabismus surgery. Accordingly, we have reconsidered the importance and benefits of early surgical intervention for patients with IXT when their stereoacuity is impaired, but not completely absent.

The results of this study have several limitations. First, although relatively uniform manipulations for surgery and examination were followed in our hospital, the fact that 4 separate surgeons and 3 study examiners were used will introduce some variability in the data. Second, the retrospective nature of this study can be a source of concern. Third, the follow-up times were quite variable among individual cases and the average follow-up time was somewhat brief for evaluating long-time therapeutic outcomes. Fourth, even though exotropia onset was confirmed with photographic documentation in some patients, in many cases the onset of exotropia in this study was based on patient history, which can be subject to recall bias. Finally, our hospital mainly enrolled patients from South China, which could introduce some geographical bias in our series.

## CONCLUSION

Most patients with IXT showed normal visual acuity and ocular motility. A few patients had positive family histories, amblyopia, oblique muscle dysfunction, and dissociated vertical deviation. The most common subtype of IXT was the basic type. Surgical results were less favorable when the sensory status was used as the criteria for surgical success. Patients with stereoacuity loss are at increased risk for poor outcomes. To obtain a better outcome, early surgical intervention might be advisable when stereoacuity is impaired but not completely absent.

## References

[1] Govindan M Mohney BG Diehl NN Incidence and types of childhood exotropia. *Ophthalmology* 2005; 112:104–108. 15629828 10.1016/j.ophtha.2004.07.033

[2] Jenkins R Demographics geographic variations in the prevalence and management of exotropia. *Am Orthopt J* 1992; 42:82–87.

[3] Yu CBO Fan DSP Wong VWY Changing patterns of strabismus: a decade of experience in Hong Kong. *Br J Ophthalmol* 2002; 86:854–856. 12140202 10.1136/bjo.86.8.854 PMC1771235

[4] Buck D Powell C Cumberland P Presenting features and early management of childhood intermittent exotropia in the UK: inception cohort study. *Br J Ophthalmol* 2009; 93:1620–1624. 19605936 10.1136/bjo.2008.152975

[5] Abroms AD Mohney BG Rush DP Timely Surgery in intermittent and constant exotropia for superior sensory outcome. *Am J Ophthalmol* 2001; 131:111–116. 11162985 10.1016/s0002-9394(00)00623-1

[6] Hatt SR Gnanaraj L Interventions for intermittent exotropia. *Cochrane Database Syst Rev* 2013; 5: CD003737. 10.1002/14651858.CD003737.pub3 PMC4307390 23728647

[7] Pineles SL Ela-Dalman N Zvansky AG Long-term results of the surgical management of intermittent exotropia. *J AAPOS* 2010; 14:298–304. 20736121 10.1016/j.jaapos.2010.06.007

[8] Wang L Wu Q Kong X Comparison of bilateral lateral rectus recession and unilateral recession resection for basic type intermittent exotropia in children. *Br J Ophthalmol* 2013; 97:870–873. 23645821 10.1136/bjophthalmol-2013-303167

[9] Fiorelli VM Goldchmit M Uesugui CF Intermittent exotropia: comparative surgical results of lateral recti-recession and monocular recess-resect. *Arq Bras Oftalmol* 2007; 70:429–432. 17768548 10.1590/s0004-27492007000300008

[10] Saleem QA Cheema AM Tahir MA Outcome of unilateral lateral rectus recession and medial rectus resection in primary exotropia. *BMC Res Notes* 2013; 6:257. 23834953 10.1186/1756-0500-6-257 PMC3708763

[11] Yang X Man TT Tian QX Long-term postoperative outcomes of bilateral lateral rectus recession vs unilateral recession-resection for intermittent exotropia. *Int J Ophthalmol* 2014; 7:1043–1047. 25540763 10.3980/j.issn.2222-3959.2014.06.25 PMC4270974

[12] Jang JH Park JM Lee SJ Factors predisposing to consecutive esotropia after surgery to correct intermittent exotropia. *Graefes Arch Clin Exp Ophthalmol* 2012; 250:1485–1490. 22450527 10.1007/s00417-012-1991-y

[13] Kim HJ Choi DG Consecutive esotropia after surgery for intermittent exotropia: the clinical course and factors associated with the onset. *Br J Ophthalmol* 2014; 98:871–875. 24627254 10.1136/bjophthalmol-2013-304726

[14] Lim SH Hwang BS Kim MM Prognostic factors for recurrence after bilateral rectus recession procedure in patients with intermittent exotropia. *Eye* 2012; 26:846–852. 22441025 10.1038/eye.2012.55 PMC3376299

[15] Jae-Wook Jung Se-Youp Lee A Comparison of the clinical characteristics of intermittent exotropia in children and adults. *Korean J Ophthalmol* 2010; 24:96–100. 20379459 10.3341/kjo.2010.24.2.96 PMC2851009

[16] Yam JC Wu PK Chong GS Long-term ocular alignment after bilateral lateral rectus recession in children with infantile and intermittent exotropia. *J AAPOS* 2012; 16:274–279. 22681946 10.1016/j.jaapos.2012.01.005

[17] Nusz KJ Mohney BJ Diehl N Female predominance in intermittent exotropia. *Am J Ophthalmol* 2005; 140:546–547. 16139014 10.1016/j.ajo.2005.03.026

[18] Park KH Kim SY Clinical characteristics of patients that experience different rates of exodrift after strabismus surgery for intermittent exotropia and the effect of the rate of exodrift on final ocular alignment. *J AAPOS* 2013; 17:54–58. 23415036 10.1016/j.jaapos.2012.10.014

[19] Mohney B Huffaker RK Common forms of childhood exotropia. *Ophthalmology* 2003; 110:2093–2096. 14597514 10.1016/j.ophtha.2003.04.001

[20] Wilson ME Parks MM Primary inferior oblique overaction in congenital esotropia, accommodative esotropia, and intermittent exotropia. *Ophthalmology* 1989; 96:950–957. 2771362 10.1016/s0161-6420(89)32774-6

[21] Struck MC Daley TJ Resolution of hypertropia with correction of intermittent exotropia. *Br J Ophthalmol* 2013; 97:1322–1324. 23934140 10.1136/bjophthalmol-2013-303553

[22] Choi J Chang JW Kim SJ The long-term survival analysis of bilateral lateral rectus recession versus unilateral recession-resection for intermittent exotropia. *Am J Ophthalmol* 2012; 153:343–351. 21982103 10.1016/j.ajo.2011.06.024

[23] Jeoung JW Lee MJ Hwang JM Bilateral lateral rectus recession versus unilateral recess-resect procedure for exotropia with a dominant eye. *Am J Ophthalmol* 2006; 141:683–688. 16564803 10.1016/j.ajo.2005.11.021

[24] Ekdawi NS Nusz KJ Diehl NN Postoperative outcomes in children with intermittent exotropia from a population-based cohort. *J AAPOS* 2009; 13:4–7. 18848478 10.1016/j.jaapos.2008.06.001 PMC2762935

[25] Isenberg SJ Abdarbashi P Drift of ocular alignment following strabismus surgery. Part 2: using adjustable sutures. *Br J Ophthalmol* 2009; 93:443–447. 18653587 10.1136/bjo.2007.136382

[26] Chiu AK Din N Ali N Standardising reported outcomes of surgery for intermittent exotropia—a systematic literature review. *Strabismus* 2014; 22:32–36. 24564726 10.3109/09273972.2013.877940

[27] Raab EL Parks MM Recession of the lateral recti: Early and late postoperative alignments. *Arch Ophthalmol* 1969; 82:203–208. 5796092 10.1001/archopht.1969.00990020205010

[28] Oh JY Hwang JM Survival analysis of 365 patients with exotropia after surgery. *Eye* 2006; 20:1268–1272. 16167074 10.1038/sj.eye.6702091

[29] Choi J Kim SJ Yu YS Initial postoperative deviation as a predictor of long-term outcome after surgery for intermittent exotropia. *J AAPOS* 2011; 15:224–229. 21665502 10.1016/j.jaapos.2010.12.019

[30] Pineles SL Deitz LW Velez FG Postoperative outcomes of patients initially overcorrected for intermittent exotropia. *J AAPOS* 2011; 15:527–531. 22153394 10.1016/j.jaapos.2011.08.007 PMC3713806

